# Hematological Indexes Can Be Used to Predict the Incidence of Hypothyroidism in Nasopharyngeal Carcinoma Patients after Radiotherapy

**DOI:** 10.1155/2020/3860936

**Published:** 2020-05-11

**Authors:** Ling Zhou, Jia Chen, Chang-Juan Tao, Shuang Huang, Jiang Zhang, Wei Shen, Chao-Nan Zhu, Ming Chen, Zhong-Hua Yu, Yuan-Yuan Chen

**Affiliations:** ^1^Institute of Cancer and Basic Medical (ICBM), Chinese Academy of Sciences, Hangzhou, China; ^2^Department of Radiation Oncology, Cancer Hospital of University of Chinese Academy of Sciences, Hangzhou, China; ^3^Department of Radiation Oncology, Zhejiang Cancer Hospital, Hangzhou, China; ^4^Postgraduate Education, Guangdong Medical University, Zhanjiang, China; ^5^Hangzhou YITU Healthcare Technology Co., Ltd., Hangzhou, China; ^6^Department of Oncology, The Affiliated Hospital of Guangdong Medical University, Zhanjiang, China

## Abstract

**Background:**

This study explored the relationship between thyroid-associated antibodies, immune cells, and hypothyroidism to establish a predictive model for the incidence of hypothyroidism in patients with nasopharyngeal carcinoma (NPC) after radiotherapy.

**Methods:**

A total of 170 patients with NPC treated at the Cancer Hospital of University of Chinese Academy of Sciences between January 2015 and August 2018 were included. The complete blood count, biochemical, coagulation function, immune cells, and thyroid-associated antibodies tested before radiotherapy were evaluated. A logistic regression model was performed to elucidate which hematological indexes were related to hypothyroidism development. A predictive model for the incidence of hypothyroidism was established. Internal verification of the multifactor model was performed using the tenfold cross-validation method.

**Results:**

The univariate analysis showed that immune cells had no statistically significant differences among the patients with and without hypothyroidism. Sex, N-stage, antithyroid peroxidase antibody (TPO-Ab), antithyroglobulin antibody (TG-Ab), thyroglobulin (TG), and fibrinogen (Fb) were associated with hypothyroidism. Males and early N-stage were protective factors of thyroid function, whereas increases in TPO-Ab, TG-Ab, TG, and Fb counts were associated with an increased rate of hypothyroidism incidence. The multivariate analysis showed that TPO-Ab, TG-Ab, TG, and Fb were independent predictors of hypothyroidism. The comprehensive effect of the significant model, including TPO-Ab, TG-Ab, TG, and Fb counts, represented the optimal method of predicting the incidence of radiation-induced hypothyroidism (AUC = 0.796). Tenfold cross-validation methods were applied for internal validation. The AUCs of the training and testing sets were 0.792 and 0.798, respectively.

**Conclusion:**

A model combining TPO-Ab, TG-Ab, TG, and Fb can be used to screen populations at a high risk of developing hypothyroidism after radiotherapy.

## 1. Background

Nasopharyngeal carcinoma (NPC) is one of the common malignant tumors in Southern China and Southeast Asia. Moreover, NPC is associated with previous Epstein-Barr virus (EBV) infection, for which radiotherapy is the primary treatment. Due to the development of intensity-modulated radiation therapy (IMRT) and comprehensive therapy, the five-year survival rate can be as high as 80% [1, 2]. In addition, with the extension of survival time, patient quality of life has been an issue of increasing attention. IMRT can increase the dose of radiation to the tumor target area and reduce the incidence of side effects in the surrounding normal tissues [3]. However, due to their special anatomical structure, the pituitary and thyroid glands will inevitably be exposed to a dose of radiation, which results in radiation-induced hypothyroidism. Hypothyroidism is a pathological condition resulting from thyroid hormone deficiency, which is divided into clinical and subclinical hypothyroidism. The incidence of hypothyroidism after radiotherapy increased from 20% to 60% during the era of IMRT treatment and could not be effectively controlled [4, 5]. The most common symptoms of hypothyroidism consisted of fatigue, drowsiness, fear of cold, weight gain, constipation, and dry skin. In severe cases, it can even lead to heart disease, including coronary heart disease, heart failure, and other conditions [6]. Therefore, even during IMRT treatment, greater attention should be paid regarding the side effects on the thyroid glands after radiotherapy.

Recently, a large number of studies have reported that the immune system plays a key role in radiation response [7, 8], which is divided into adaptive and innate immunity. Adaptive immunity consists primarily of B and T lymphocytes. B lymphocytes are the precursors of plasma cells and are regulated by T lymphocyte subsets. T lymphocyte subsets are one of the most important cell groups in the immune system and can be divided into CD4+ and CD8+ T cell populations. Natural killer (NK) cells are a type of innate immune cell that can activate the adaptive immune system via critical signals. Thus, T lymphocytes, B lymphocytes, and NK cells are the key mediators of the radiation-induced immune response. Moreover, evidence from other studies has demonstrated reduced efficacy for radiotherapy of patients who aredeficient in immune cells [9, 10]. Currently, although the mechanism of radiation-induced hypothyroidism remains unclear, it is generally believed that radiation-induced immune response is one of the main mechanisms [11, 12]. Some previous studies have demonstrated that the concentration of thyroid-associated antibodies (i.e., antithyroid peroxidase antibody (TPO-Ab) and antithyroglobulin antibody (TG-Ab)) can be correlated with hypothyroidism [13]. Moreover, the incidence of hypothyroidism is higher in patients positively expressing TPO-Ab and TG-Ab [14]. However, no relevant prediction model based on immune indicators has been established to predict the incidence of radiation-induced hypothyroidism in NPC. Therefore, this study is aimed at exploring the relationship between thyroid-associated antibodies, immune cells, and the incidence rate of hypothyroidism. The purpose of such findings was to explain the internal relationship between radiation-induced hypothyroidism and immune function, as well as establish a model with the relevant hematological indexes to predict the incidence rate of hypothyroidism in patients with NPC after radiotherapy.

## 2. Methods

### 2.1. Patient Selection

Patients with NPC treated at the Cancer Hospital of University of Chinese Academy of Sciences from January 2015 to August 2018 were included in the present study. The eligibility criteria for this study included patients with NPC diagnosed by pathology, who had complete medical records, and thyroid-associated antibody and immune function tests prior to radiotherapy. The follow-up period for the thyroid function test was greater than one year for all of the patients. Patients with immune system, hypothalamus, pituitary, or thyroid diseases were excluded from this study.

### 2.2. Radiotherapy and Chemotherapy

#### 2.2.1. Chemotherapy

All patients received 2-3 cycles TP (docetaxel/paclitaxel+cisplatin/nedaplatin), PF (cisplatin/nedaplatin+fluorouracil), or TPF (docetaxel/paclitaxel+cisplatin/nedaplatin + fluorouracil)-induced chemotherapy. Chemotherapy with cisplatin or nedaplatin was performed simultaneously during radiotherapy.

#### 2.2.2. Radiotherapy

All of the patients received IMRT. The specific doses assigned to the gross primary tumor, positive lymph nodes, high-risk areas, and low-risk areas were 66 Gy-74 Gy, 60 Gy-70 Gy, 60 Gy-62 Gy, and 50 Gy-56 Gy, respectively, which were divided into 33 fractions. The high-risk areas included the primary nasopharyngeal tumor and mucosa, and the low-risk areas included the posterior maxillary sinus, pterygopalatine fossa, parapharyngeal space, skull base, part of posterior ethmoid sinus, and bilateral lymphatic drainage area. The limit of endangering organs was subjected to the established guidelines [15].

### 2.3. Hematological Indexes

The complete blood count, biochemical, coagulation function, immune cell, and thyroid-associated antibody tests before radiotherapy were evaluated for all of the patients. The complete blood count included the white blood cells, neutrophils, lymphocytes, monocytes, eosinophils, basophils, red blood cells, platelets, and hemoglobin. Biochemical tests included the albumin, globulin, total protein, lipoprotein, alanine aminotransferase, aspartate aminotransferase, cholinesterase, creatinine, bilirubin, bile acid, uric acid, and glucose. The coagulation function test included the partial thromboplastin time, prothrombin time, and fibrinogen (Fb) content. The immune cells included T cells, B cells, and NK cells. The thyroid-associated antibodies included TPO-Ab, TG-Ab, and thyroglobulin (TG).

### 2.4. Examination of Thyroid Function

All patients underwent a thyroid function test before and after radiotherapy, which included thyroid-stimulating hormone (TSH), triiodothyronine (FT_3_), and free thyroxine (FT_4_) using the electrochemiluminescence method with the SIEMENS ADVIA Centaur XP, with a reference range of TSH and FT_4_ being 0.380-4.340 IU/mL and 0.81-1.89 ng/dL, respectively. Biochemical hypothyroidism was defined as TSH concentrations above the reference range and FT_4_ concentrations within or lower than the normal range [12]. The above definitions cover both clinical and subclinical hypothyroidism. Central hypothyroidism was defined as low FT4 levels with low or low-to-normal levels of TSH. After radiotherapy, thyroid function tests were performed at least once every six months.

### 2.5. Statistical Analysis

Firstly, the clinical characteristics stratified by hypothyroidism were described using the means and standard deviations (for normally distributed variables), interquartile range (median (Q25-Q75)) (for abnormally distributed variables), or frequency and percentages (for categorical variables). Their differences were then correspondingly compared with a *t*-test, Wilcoxon rank sum test (or Mann-Whitney *U* test), and Chi-square test (or Fisher's exact probability), respectively. Secondly, a series of univariate logistic regression models were performed to examine which of the clinical and immunological indexes were related to the development of hypothyroidism. Only the characteristics that were significantly different in the univariate analysis were included in the logistic regression model, and the independent predictors of hypothyroidism were identified using backward elimination (*P* > 0.1 was excluded). Moreover, the internal verification of the multifactor model was performed using the tenfold cross-validation method. Thirdly, the cut-off for the immunological index was calculated using a receiver operating characteristics (ROC) curve. Finally, we established a joint prediction model according to the cut-offs of the significantly independent variables. *P* < 0.05 was considered to be statistically significant. All analyses were performed using R-3.6.0.

## 3. Results

### 3.1. Patient Characteristics

The median follow-up time of the 170 patients was 20 months (13-36 months) and the incidence rate of primary hypothyroidism was 45.29% (77/170); no patients were diagnosed with central hypothyroidism. The average age of the patients was 51.55 ± 10.55 years old, including 115 males and 55 females. The patients' general clinical characteristics and the distribution of immune cells are listed in Table 1.

### 3.2. Factors Related to Radiation-Induced Hypothyroidism

The univariate analysis demonstrated that sex, N-stage, and pretreatment TPO-Ab, TG-Ab, TG, and Fb counts were associated with hypothyroidism. Among these factors, males and early N-stage were protective factors of thyroid function, whereas the higher the pretreatment TPO-Ab, TG-Ab, TG, and Fb counts, the higher the hypothyroidism incidence rate. No differences were shown for the factors of age, clinical stage, chemotherapy, and other hematological indicators among the patients with and without hypothyroidism. The multivariate analysis showed that the pretreatment TPO-Ab, TG-Ab, TG, and Fb counts were independent predictors of hypothyroidism (Table 2).

The cut-off for the immunological index was calculated using the ROC, and the optimal TPO-Ab, TG-Ab, TG, and Fb counts were determined using the maximum Youden index (specificity + sensitivity − 1). Patients were then accordingly reclassed into two groups with respect to the specific cut-off value; the critical values of TPO-Ab, TG-Ab, TG, and Fb related to the incidence of radiation-induced hypothyroidism were 7.40 IU/mL (normal value: 0-34 IU/mL), 57.40 IU/mL (normal value: 0-115 IU/mL), 16.65 ng/mL (normal value: 1.40-78 ng/mL), and 3.50 g/L (normal value: 2-4 g/L), respectively. The incidence rates of hypothyroidism were 11.11% vs. 51.75%, 37.21% vs. 70.73%, 37.88% vs. 71.05%, and 36.45% vs. 60.32%, respectively (Table 3).

### 3.3. Prediction Model

The prediction models were built according to the results of the univariate and multivariate analyses. A full prediction model was established by including the indexes that were statistically significant in the univariate analysis, including sex, N-stage, and TPO-Ab, TG-Ab, TG, and Fb counts. A significant model was built by including indexes with significant statistical significance in the multivariate analysis, including TPO-Ab, TG-Ab, TG, and Fb counts (Table 4).

**Figure 1:**
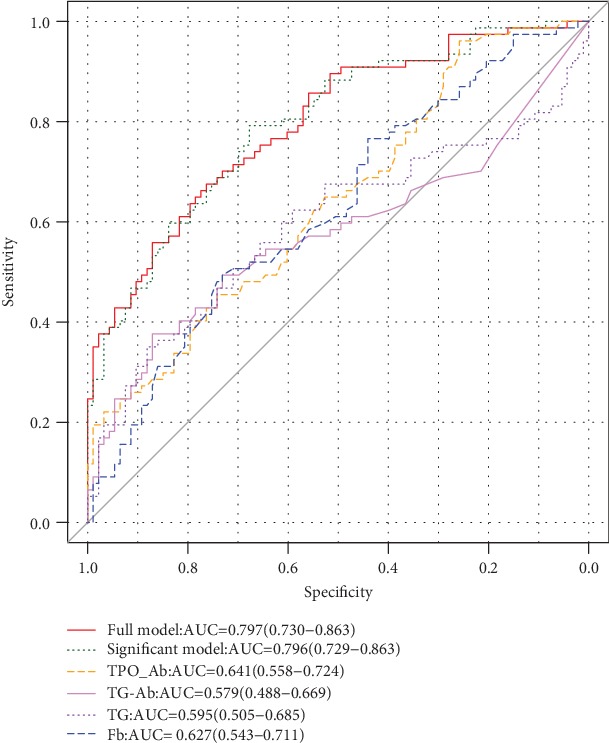
Analysis of the area under the ROC curve in patients with NPC treated with IMRT.

The ROC curves showed that the effect of combining TPO-Ab, TG-Ab, TG, and Fb with the four indexes to predict the incidence of hypothyroidism was significantly higher than that of their independent prediction (AUC = 0.796). The combination of all relevant factors was used to establish a full model to predict the incidence of hypothyroidism (AUC = 0.797) (Figure 1).

**Figure 2:**
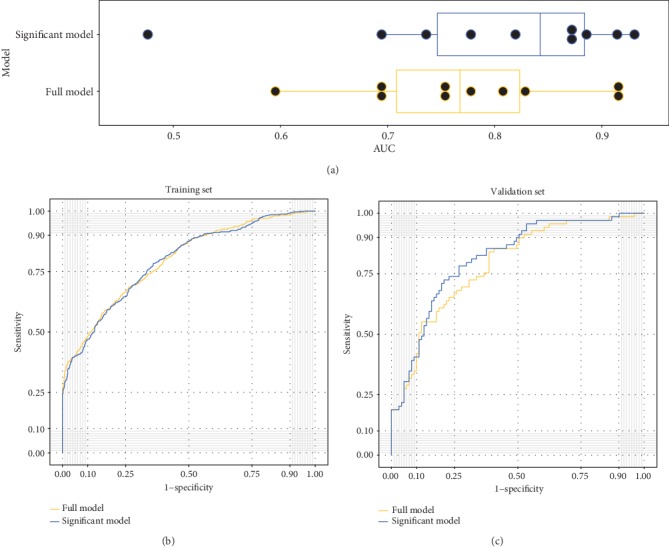
Predictive power comparison between the full model and significant model under multivariate analysis. (a) Box plot of AUC distribution of the 10 logistic regression models in each tenfold cross-validation. (b) Training ROC curves for the full model (red) and significant model (blue). The AUCs of the two curves are 0.794 and 0.792, respectively. (c) Testing ROC curves for the full model (red) and significant model (blue). The AUCs of the two curves are 0.774 and 0.798, respectively.

Furthermore, a tenfold cross-validation method was used to verify the multifactor model built with the full model and significant model. The AUCs of the training set for the full model and the significant model were 0.794 and 0.792, respectively, and the AUCs of the testing set of the full model and the significant model were 0.774 and 0.798, respectively (Figure 2). Therefore, the comprehensive effect of the significant model represented the best method of predicting the incidence of radiation-induced hypothyroidism, which showed 79.2% sensitivity and 67.7% specificity.

## 4. Discussion

Hypothyroidism is a common complication of nasopharyngeal carcinoma after radiotherapy. In our study, the incidence of biochemical hypothyroidism was 45.29%, and no patients were diagnosed with central hypothyroidism, which may be due to insufficient follow-up time. Bhandare et al. showed that the incidence of central hypothyroidism was 5.4%, and the median latency period was 4.8 years [16].

The thyroid gland is the largest endocrine gland in the body, which primarily secretes thyroid hormones and its insufficient secretion causes hypothyroidism. The clinical manifestations of hypothyroidism vary according to age and sex, with mild cases being asymptomatic, severe cases being life-threatening, and a mortality rate as high as 40% [17]. Currently, the pathogenesis of hypothyroidism remains poorly understood. Studies have shown that hypothyroidism is closely related to abnormal lipid metabolism, cardiovascular, autoimmune, and other diseases [18, 19].

It is now believed that radiation-induced hypothyroidism in patients with NPC is significantly related to the radiation dose, age, sex, and clinical stage. Sommat et al. [20] considered that a younger age and larger T-stage were risk factors of hypothyroidism, whereas a thyroid V_40_  ≤  85% was a protective factor for thyroid function. Moreover, the retrospective analysis performed by Ling et al. [21] showed that thyroid V_50_ was an important predictor of radiation-induced hypothyroidism, and thyroid V_50_  <  50% was closely related to the incidence of hypothyroidism. In addition, Zhai et al. [22] recommended that the thyroid dose be set to V_50_  ≤  35% and V_45_  ≤  50% to protect thyroid function. Since there is no unified standard for the optimal dose limit of the thyroid gland, several studies have explored this topic, while ignoring the internal relationship between multiple systems in the body with hypothyroidism, which is an endocrine disease.

### 4.1. Thyroid-Associated Antibodies Associated with Hypothyroidism

The most common cause of hypothyroidism is autoimmune disease, and a large number of studies have shown that TPO-Ab and TG-Ab are closely related to hypothyroidism [13]. Moreover, patients positive for TPO-Ab and TG-Ab are more likely to develop hypothyroidism [23], and their concentrations can predict whether subclinical hypothyroidism will develop into clinical hypothyroidism [14]. This is consistent with our findings which showed that TG, TG-Ab, and TPO-Ab are independent predictors of hypothyroidism. In addition, TG is primarily synthesized and secreted by thyroid follicular cells and regulated by TSH. As an autoantibody produced by TG, the higher the level of TG-Ab expression, the more serious the immune function disorder [24]. TPO is not only a key enzyme involved in thyroid hormone synthesis but also an important antigen responsible for causing autoimmune thyroid disease. Moreover, the abnormal expression of TPO and the immune response associated with TPO-Ab are important mechanisms of thyroid cell injury in patients with hypothyroidism. After radiotherapy, thyroid inflammation occurs in patients with nasopharyngeal carcinoma, which results in increased TG-Ab and TPO-Ab expression. Such expression impacts the synthesis of thyroid hormones and leads to hypothyroidism. Based on the information published in previous studies, we found that the increased expressions of TG, TG-Ab, and TPO-Ab in the serum are important predictors of hypothyroidism. In addition, these predictors are also combined with all independent predictors to establish a model for predicting the incidence of hypothyroidism with a favorable prediction efficiency. Moreover, cross-validation was performed internally, which can be just as effective as using a separate test set to estimate the generalization error of the training set.

Although several studies have shown that hypothyroidism is related to TPO-Ab, TG-Ab, and TG, few studies have investigated the relationship between radiation-induced hypothyroidism and thyroid-associated antibodies. Lin et al. [25] believed that there was a strong correlation between the levels of thyroid hormones and thyroid-associated antibodies in patients with NPC after radiotherapy; however, they did not further explore the level of thyroid-associated antibody expression related to hypothyroidism. Our study is the first to explore the correlation between NPC, radiotherapy, immunity, and hypothyroidism. The level of thyroid-associated antibody expression, immune cells, and coagulation function indexes before radiotherapy were used to screen high-risk patients with hypothyroidism. We found that the level of thyroid-associated antibody expression and fibrinogen were independent predictors of radiation-induced hypothyroidism. It was additionally shown that the significant model combined with TPO-Ab, TG-Ab, TG, and Fb was the optimal method used to predict the incidence of hypothyroidism after radiotherapy. The model was internally verified to exhibit a good performance using the tenfold cross-validation method. The cut-off of TPO-Ab and TG-Ab for hypothyroidism after radiotherapy is 7.40 IU/mL and 57.40 IU/mL, respectively, which is much less than the lower limit of normal. Together, these findings suggest that immunity plays an important role in thyroid function damage caused by radiotherapy.

### 4.2. Immune Cells Associated with Hypothyroidism

Over the past century, there have been multiple studies on the humoral and cellular immune mechanisms related to autoimmune thyroid diseases, especially humoral immunity [26]. Segundo et al. [27] identified a class of B lymphocytes (IgM-, IgD-, CD44-, CD38++, CD71+, and CD95+) in the thyroid gland that was closely related to the concentration of TPO-Ab in the serum. Moreover, the CD4+ to CD8+ T cell ratio is also an important cause of autoimmune disease. The purpose of this study was to analyze the relationship between T cell subsets (CD3+, CD4+, CD8+, and CD4+/CD8+), B cells (CD19+), and NK cells (CD56+) with radiation-induced hypothyroidism. No differences were identified among the patients with and without hypothyroidism, which may be due to the complex relationship between radiotherapy, immunity, and thyroid function that is disturbed by other factors. Since this study was the first to explore the correlation between radiation hypothyroidism and immune function, future research should eliminate some interference factors and explore the mechanism between radiation hypothyroidism and immune function in greater depth.

### 4.3. Fb Associated with Hypothyroidism

Previous studies have shown that patients with hypothyroidism often have risk factors for cardiovascular disease (e.g., hypertension, high cholesterol, and hyperlipidemia) [28, 29]. We found that the fibrinogen content in high-risk patients with hypothyroidism was significantly higher than that of normal patients. Moreover, fibrinogen is an important factor in atherosclerosis and thrombosis. In this study, most patients with hypothyroidism also had high levels of fibrinogen, exhibiting hypercoagulable blood. This is consistent with the results reported by Ordookhani and Burman [30], who considered the increased coagulation factor VII and plasminogen activator inhibitor-1 activity to be one of the main mechanisms leading to the hypercoagulable state in patients with hypothyroidism.

### 4.4. Other Factors Associated with Hypothyroidism

The neutrophil-to-lymphocyte ratio in the serum is a common prognostic indicator, which is often used to predict the therapeutic toxicity, systemic inflammation, and tissue damage of various cancers [31–33]. In this study, we also aimed to explore the correlation between the neutrophil-to-lymphoid ratio and radiation-induced hypothyroidism. However, the neutrophil-to-lymphoid ratio did not appear to predict the incidence of hypothyroidism in patients with NPC after radiotherapy.

The purpose of this study was to screen outpatients at a high risk of hypothyroidism who were sensitive to radiotherapy in an effort to prompt clinicians to closely monitor the thyroid function of high-risk patients after radiotherapy. Although interfering factors were excluded as much as possible, there is an inevitability of confounding factors associated with the retrospective analysis. There are some shortcomings associated with our study, including the retrospective nature and the median follow-up time of only 20 months. This may also be the reason why no central hypothyroidism was observed in this study. In the future, further prospective studies should be performed to explore the relationship between radiotherapy, immunity, and hypothyroidism in patients with NPC.

## 5. Conclusions

This study is the first to explore the relationship between hematological indexes (e.g., thyroid-associated antibodies, immune cells, coagulation function, and radiation-induced hypothyroidism) in patients with NPC. TPO-Ab, TG-Ab, TG, and Fb were found to represent independent predictors of radiation hypothyroidism. Thus, a significant model combined with TPO-Ab, TG-Ab, TG, and Fb may be used to screen high-risk populations for hypothyroidism after radiotherapy (AUC = 0.796).

## Figures and Tables

**Table 1:** Distribution of general clinical features and immunological indexes in 170 patients with NPC after IMRT.

Variables	170 patients with NPC after IMRT		Statistic	*P* value
	Without hypothyroidism (*N* = 93)	With hypothyroidism (*N* = 77)		
Age (mean (SD))	52.66 (10.84)	50.22 (10.1)	*t* = 1.50	0.1347
Sex			*χ* ^2^ = 5.4499	0.0196^∗^
Male	70 (41.18)	45 (26.47)		
Female	23 (13.53)	32 (18.82)		
T-stage (2010UICC)			*χ* ^2^ = 0.6396	0.4239
T_1-2_	16 (9.41)	17 (10)		
T_3-4_	77 (45.29)	60 (35.29)		
N-stage (2010UICC)			*χ* ^2^ = 4.5349	0.0332^∗^
N_0-1_	40 (23.53)	21 (12.35)		
N_2-3_	53 (31.18)	56 (32.94)		
M-stage (2010UICC)				0.3025^a^
M_0_	90 (52.94)	71 (41.76)		
M_1_	3 (1.76)	6 (3.53)		
Clinical stage (2010UICC)			*χ* ^2^ = 2.0053	0.1567
I-III	43 (25.29)	44 (25.88)		
IV	50 (29.41)	33 (19.41)		
Neoadjuvant chemotherapy				0.5141^a^
No	6 (3.53)	3 (1.76)		
Yes	87 (51.18)	74 (43.53)		
BMI				0.4927^a^
<18.5	6 (3.53)	2 (1.18)		
~24.9	64 (37.65)	53 (31.18)		
≥25	23 (13.53)	22 (12.94)		
TPO-Ab	12.5 (7.4-17.3)	14.6 (11.1-28.1)	*Z* = 3.1572	0.0016^∗∗^
TG-Ab	11.2 (10.2-16)	14.1 (10.1-151.2)	*Z* = 1.7728	0.0763
TG	7.5 (4.56-14.18)	11.95 (4.8-22.36)	*Z* = 2.1271	0.0334^∗^
CD19+	7.8 (4.8-10.9)	7.8 (5.7-11)	*Z* = 0.5964	0.5509
CD3+	69.8 (60-77.5)	69.6 (57.48-77.6)	Z = -0.6934	0.4881
CD4+	36.8 (28.9-44.3)	37.27 (30.1-42.5)	Z = 0.1315	0.8954
CD8+	26.13 (20.6-33.9)	25.2 (19.8-32.4)	Z = -0.8061	0.4202
CD4+/CD8+	1.4 (0.9-1.9)	1.4 (1.1-2.1)	*Z* = 0.7255	0.4681
CD38+	7 (4.5-9.4)	7.02 (4.7-9.32)	*Z* = 0.2129	0.8314
CD45RA-	10.2 (7.1-14.1)	9.4 (7-13.9)	*Z* = −0.4383	0.6612
CD45RA+	22 (17.67-25.6)	23.3 (17.15-28.33)	*Z* = 0.8437	0.3989
CD45RO+	22.1 (16.6-25.7)	22.13 (17.2-27.9)	*Z* = 1.1489	0.2506
CD45RA+/CD45RO+	2.7 (1.9-3.8)	2.95 (1.9-3.9)	*Z* = 0.4885	0.6252
CD56+	18.45 (12.3-27.5)	20.3 (12.8-27.17)	*Z* = 0.659	0.5099
NET cell	2.8 (1.8-5)	3.2 (1.6-4.6)	*Z* = −0.1127	0.9103
Neutrophils/lymphocytes	2.35 (1.55-4)	3 (1.42-5.3)	*Z* = 0.9251	0.3549
EBV DNA	0 (0-500.99)	0 (0-737.08)	*Z* = −0.0033	0.9974
Cholinesterase	7508 (6620-8730)	7737 (6776-9406)	*Z* = 1.3211	0.1865
Fb	3.17 (2.58-3.54)	3.5 (3.03-3.91)	*Z* = 2.8379	0.0045^∗^

Note: the values in the table are *N* (%) or median (Q_25_-Q_75_) if there is no special marking; ^a^*P* value is calculated by Fisher's exact probability. ^∗^*P* < 0.05; ^∗∗^*P* < 0.01; and ^∗∗∗^*P* < 0.001.

**Table 2:** Logistic regression analysis of radiation-induced hypothyroidism in 170 patients with NPC.

Variables (reference group)	Univariate analysis		Multivariate analysis	
	OR (95% CI)	*P* value	OR (95% CI)	*P* value
Age	0.98 (0.95-1.01)	0.1354		
Sex male (versus female)	0.46 (0.24-0.89)	0.0206^∗^	0.73 (0.33-1.61)	0.4342
BMI underweight (versus normal)	0.4 (0.08-2.08)	0.2396		
BMI overweight (versus normal)	1.16 (0.58-2.3)	0.2438		
T-stage T_2-3_ (versus T_0-1_)	0.73 (0.34-1.57)	0.4248		
N-stage N_2-3_ (versus N_0-1_)	2.01 (1.05-3.85)	0.0344^∗^	1.34 (0.63-2.85)	0.4527
M-stage M_1_ (versus M_0_)	2.54 (0.61-10.49)	0.1993		
Clinical stage IV (versus I-III)	0.65 (0.35-1.19)	0.1576		
Neoadjuvant chemotherapy (yes (versus no))	1.7 (0.41-7.04)	0.4635		
TPO-Ab	1.014 (1.003-1.026)	0.0144^∗^	1.01 (1.002-1.03)	0.025^∗^
TG-Ab	1.004 (1.001-1.006)	0.0024^∗∗^	1.004 (1.001-1.01)	0.0112^∗^
TG	1.04 (1.01-1.07)	0.0105^∗^	1.06 (1.02-1.1)	0.0009^∗∗∗^
CD19+	1.01 (0.94-1.09)	0.7506		
CD3+	0.99 (0.97-1.02)	0.4777		
CD4+	1.00 (0.97-1.03)	0.9681		
CD8+	0.98 (0.95-1.02)	0.3422		
CD4+/CD8+	1.16 (0.82-1.63)	0.4024		
CD38+	0.99 (0.92-1.06)	0.7707		
CD45RA-	1.02 (0.97-1.06)	0.5055		
CD45RA+	1.00 (0.95-1.06)	0.9631		
CD45RO+	1.03 (0.98-1.07)	0.2625		
CD45RA+/CD45RO+	1.05 (0.91-1.21)	0.5254		
CD56+	1.01 (0.98-1.04)	0.5395		
Neutrophils/lymphocytes	1.02 (0.96-1.08)	0.4844		
EBV DNA	1 (1-1)	0.3799		
Cholinesterase	1 (1-1)	0.1324		
Fb	1.88 (1.21-2.9)	0.0046^∗∗^	1.93 (1.17-3.17)	0.0098^∗∗^

Note: ^∗^*P* < 0.05; ^∗∗^*P* < 0.01; and ^∗∗∗^*P* < 0.001.

**Table 3:** The critical value of related factors with radiation-induced hypothyroidism.

Variables		Hypothyroidism		*χ* ^2^	*P* value
		No	Yes		
TPO-Ab (IU/mL)	≤7.40	24	3	15.1364	<0.001^∗∗∗^
	>7.40	69	74		

TG-Ab (IU/mL)	≤57.40	81	48	14.1097	<0.001^∗∗∗^
	>57.40	12	29		

TG (ng/mL)	≤16.65	82	50	13.1046	<0.001^∗∗∗^
	>16.65	11	27		

Fb (g/L)	≤3.50	68	39	9.1172	0.003^∗∗^
	>3.50	25	38		

Note: ^∗∗^*P* < 0.01; ^∗∗∗^*P* < 0.001.

**Table 4:** Prediction model coefficient.

Variables (reference group)	^a^Full model		^b^Significant model		TPO-Ab		TG-Ab		TG		Fb	
	Coefficient	*P* value	Coefficient	*P* value	Coefficient	*P* value	Coefficient	*Pv*alue	Coefficient	*P* value	Coefficient	*P* value
Constant	-3.9114	<.0001	-3.8895	<.0001	-0.5938	0.0027	-0.4923	0.0061	-0.664	0.0041	-2.249	0.0025
Male (versus female)	-0.1571	0.4342										
N-stage N_2-3_ (versus N_0-1_)	0.29	0.4527										
TPO-Ab	0.0134	0.025	0.0141	0.0195	0.0142	0.0144						
TG-Ab	0.00378	0.0112	0.00393	0.0086			0.0039	0.0024				
TG	0.0583	0.0009	0.061	0.0005					0.036	0.0105		
Fb	0.6561	0.0098	0.6704	0.0075							0.6287	0.0046

Note: ^a^full model: Logit (*P*/1 − *P*) = (−3.9114) + (−0.1571) × (0/1; 0 = female; 1 = male) + 0.29×((0/1; 0 = N_0−1_, 1 = N_2−3_) + 0.0134 × TPO − Ab (IU/mL) + 0.00378 × TG − Ab (IU/mL) + 0.0583 × TG (ng/mL) + 0.6561 × Fb (g/L). ^b^Significant model: Logit (*P*/1 − *P*) = (−3.8895) + 0.0141 × TPO − Ab (IU/mL) + 0.00393 × TG − Ab (IU/mL) + 0.061 × TG (ng/mL) + 0.6704 × Fb (g/L).

## Data Availability

The datasets supporting the conclusions of this article are included within the article.
